# Gold Dental Implant-Induced Oral Lichen Planus

**DOI:** 10.7759/cureus.21852

**Published:** 2022-02-02

**Authors:** Taha F Rasul, Jackson Anderson, Daniel R Bergholz, Arfa Faiz, Rishi R Prasad

**Affiliations:** 1 Infectious Diseases, University of Miami Miller School of Medicine, Miami, USA; 2 Internal Medicine, University of Miami Miller School of Medicine, Miami, USA; 3 Allergy and Immunology, University of Miami Miller School of Medicine, Miami, USA; 4 Allergy and Immunology, Sutter Medical Center, Sacramento, USA; 5 Medical Education, University of Miami Miller School of Medicine, Miami, USA

**Keywords:** mucosal irritation, heavy metal toxicity, allergy test, lichenoid reaction, atopic disease, dental implantology, dental implant, oral lichen planus

## Abstract

Lichen planus is a chronic inflammatory cutaneous and mucosal disease mostly affecting middle-aged individuals. The etiology of lichen planus is unknown, but current literature suggests that it is an altered immune response characterized by dysregulated T-cell activation and subsequent inflammation which can be associated with conditions like allergic contact dermatitis and hepatitis C. Additionally, heavy metals like lead, tin, arsenic, and bismuth can create inflammatory and allergic reactions that can predispose to the formation of lichen planus. This report examines the case of a 64-year-old female with longstanding oral lichenoid lesions with superimposed Wickham's striae, allergic skin reactions to several medications, and a history of receiving gold-containing dental implants. As a result of her history and subsequent allergy testing, she was found to have a gold allergy. The constant mucosal irritation from her dental implants likely was associated with the development of her oral lesions, which were confirmed to be oral lichen planus. She was recommended to apply triamcinolone 0.1% ointment to her oral lesions and to follow up with her dentist for evaluation of her filings. Further, it was recommended she replaces the dental crowns with compounds lacking gold to decrease the persistent irritation. This case represents the first such instance of gold dental fillings directly having an appreciable role in the development of oral lichen planus.

## Introduction

Lichen planus is a disease of chronic inflammation but of unknown origin which typically occurs in the skin and mucosa of middle-aged individuals but can occur at any age [1]. The most common lesions are purple papules having well-demarcated but irregular borders and typically occur on the distal upper and lower extremities as well as the anogenital region. One particular characteristic of the lesions is the presence of Wickham’s striae which are fine white lines occurring on the surface of the papules or plaques [2]. Although the diagnosis of lichen planus is clinical, it may be confirmed through punch biopsies or dermoscopy. Biopsy of the lesions can also help detect squamous cell carcinoma which is a potential complication of genital lichen planus. The treatment primarily includes high-dose topical corticosteroids, but oral steroids and phototherapy can be reserved for refractory cases.

The exact etiology of lichen planus is not well known. However, possible factors include an altered immune response with T-cell activation. This is why lichen planus often occurs alongside diseases like vitiligo, ulcerative colitis, and dermatomyositis which are associated with deregulated and pathological immune responses. Additionally, there is a strong association with hepatitis C infections [3]. 

Allergic contact dermatitis is a type IV hypersensitivity reaction which involves delayed T-cell sensitization and further cytotoxicity upon repeated contact. It is commonly seen with heavy metals like nickel and chromium, as well as perfumes, soaps, and certain topical medications [4]. Therefore, derangements in T-cell function could lead to downstream disease processes such as contact dermatitis and even lichen planus. 

Gold-induced lichen planus has been described as an effect of the long-term consumption of gold salts or gold-containing medications and supplements. In such cases, the eruption of lichen planus occurred not only in the oral mucosa but also in the esophagus. Our case is of a 64-year-old female with longstanding lichenoid oral lesions of uncertain etiology. 

## Case presentation

Our patient is a 64-year-old female who was being reevaluated for contact dermatitis. She was referred to the clinic in December 2021. Her medical history was notable for self-reported allergic reactions to multiple medications. Notably, she developed a maculopapular rash on the palms, soles, and chest seven days after starting doxazosin. She described developing similar reactions as well as lichenoid lesions with multiple antihypertensive medications. She complained of long-term reddish-brownish papules that would occur even after taking non-antihypertensive medications, including penicillin V, trimethoprim-sulfamethoxazole (urticarial rash), and other sulfa antibiotics. These findings were not independently reproduced or tested by an allergist.

**Figure 1:**
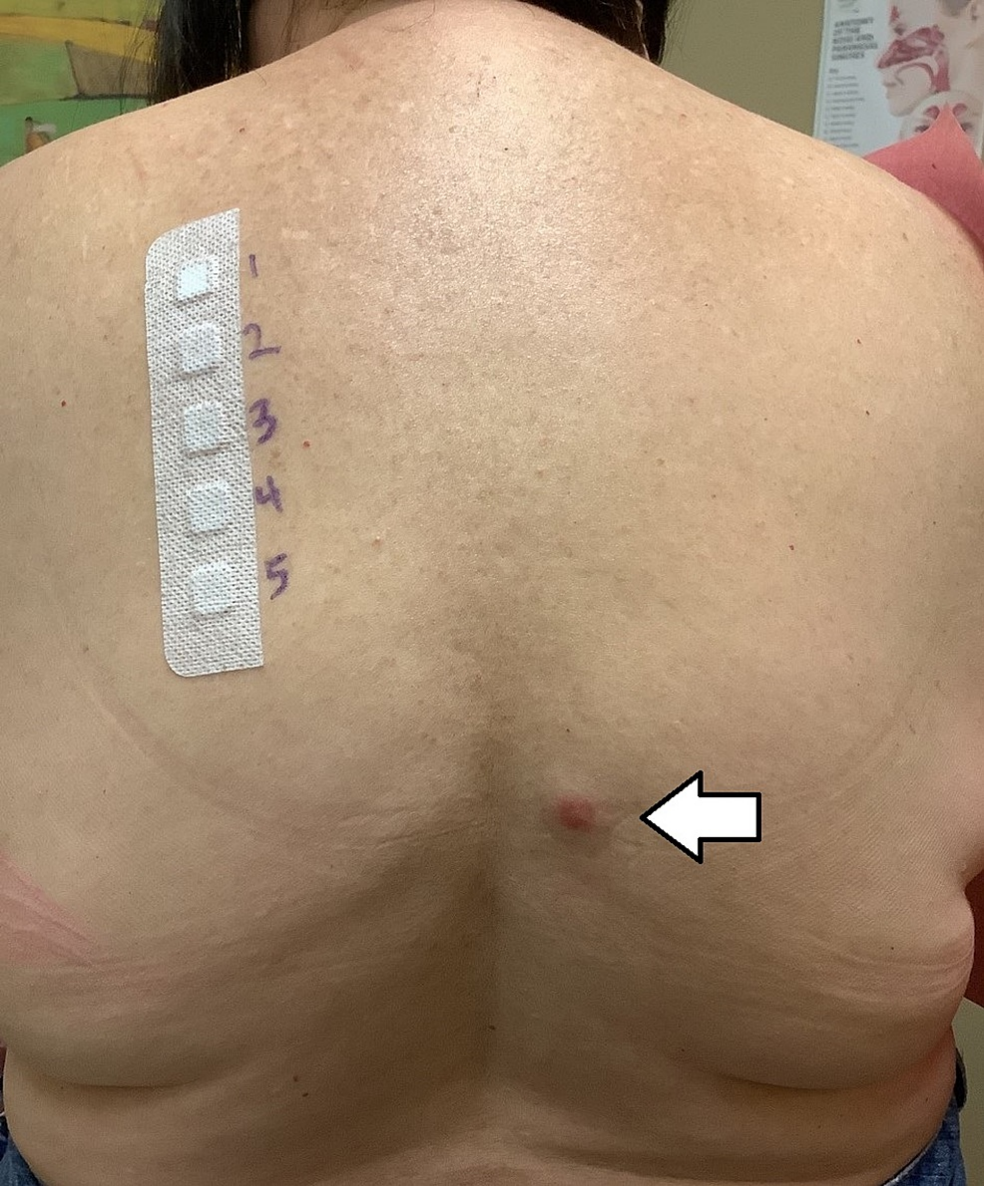
Allergy testing for various heavy metals. Erythematous papule (white arrow) denotes positive test for gold sodium thiosulfate.

She then had a broad patch-testing panel performed which showed a positive allergic reaction toward gold, specifically gold sodium thiosulfate (Figure 1). There were negative results to other common allergens and common environmental triggers like heavy metals and aromatic compounds.

Her dental conditions required the placement of multiple metal crowns. Prior to this visit, her dentist informed her of the raised, striated lesions in her mouth. Additionally, she described a history of breakouts of similar lesions on her upper extremities bilaterally which she had been treating with topical triamcinolone for several years. 

Review of systems was notable for dysphagia and rashes over her body. Upon physical examination, hyperpigmented and thickened patches were noted along the gumline on the upper and lower teeth. There were no oral ulcers. The patient's unclear severe acute respiratory syndrome coronavirus 2 (SARS-CoV-2) status necessitated prompt evaluation and remasking. Therefore, pictures were not taken of the mucosal lesions.

Her clinical presentation, as well as previous history of lichen planus, made her oral lesions likely to be a continuation of the same disease process. She was recommended to apply triamcinolone 0.1% ointment to her oral lesions and to follow up with her dentist for evaluation of her filings. At her next follow-up visit, dental analysis revealed a significant gold content within the metal bases of her crowns. Further dental follow-up to potentially replace the crowns with compounds lacking gold was recommended to the patient. 

## Discussion

Our 64-year-old patient with a history of multidrug allergic reactions and sporadic lichenoid eruptions likely had oral lichen planus secondary to longstanding gold implants. In this case, the gold dental crowns caused a local allergic reaction in the oral mucosa, which further developed into lichen planus. The generalized T-cell dysfunction seen in contact dermatitis can predispose to the development of lichen planus, which is also a delayed type of hypersensitivity. In this case, the continued localized inflammation from the gold crowns meant that there would be a constant state of immune hyperactivity. Since lichen planus is chronic and often manifests with several relapses, this could also explain our patient’s periodic breakout rashes on her upper extremities. Additionally, the Koebner phenomenon is very common, where areas of previously traumatized skin are more likely to develop the lesions [5]. This can also be why most of our patient’s oral lesions were directly proximal to the gold crowns, in areas of elevated inflammation [6].

There have been multiple cases of gold medication- or supplement-induced oral lichen planus. However, this case represents the first such case where oral lichen planus was due to the gold additives within permanent dental crowns [7]. The recommended triamcinolone cream would treat the eruptions and outbreaks but would not be useful in treating the underlying cause. Potential removal or replacement of the crowns was discussed with the patient. This would ideally remove the nidus for inflammation and hypersensitivity and decrease the likelihood of lichenoid eruptions. There could also be a partial or complete remission of the lichen planus. However, in patients with extensive dental work, this can be unfeasible due to the steep costs. In such cases, a risk-versus-benefits approach should analyze the tangible benefits of undergoing such interventions. Situations where lichen planus is likely being caused by a known allergen or sensitizing agent should involve removal of the stimulus to assess for improvement in symptoms. There have not been any major studies investigating this, and it would be an important area of research. This may also help in understanding the exact pathophysiology of lichen planus, as well as specific aggravating or relieving factors. 

A review on dental amalgam-associated oral lichenoid lesions (OLLs) and oral lichen planus (OLP) found that approximately one-third of patients experienced complete resolution, another one-third experienced partial resolution, and the remaining third had no resolution of OLP upon removal of the amalgam [8]. For patients with OLP, 89.2% of patients with positive patch tests and 78.9% of patients with negative patch tests experienced improvement with removal of the amalgam. These findings suggest that there is a likely therapeutic benefit in removal of the amalgam in most situations. A case-control study in 2003 also found that even though the presence of gold itself is not associated with an increased risk of OLP, the corrosion of amalgams can cause a "galvanic effect" which can increase the likelihood of OLP development [9].

The reactions experienced by our patient with lesions closely resembling lichen planus are likely lichenoid drug eruptions [10]. These are dermatologic conditions triggered by certain drugs or medications such as beta-blockers, angiotensin-converting enzyme (ACE) inhibitors, and penicillamine. The lesions closely resemble lichen planus but are not histologically identical. From a pathology perspective, lichen planus lesions demonstrate hyperkeratosis, wedge-shaped hypergranulosis, and irregular acanthosis (saw-tooth pattern). The lesions also have lymphocytic infiltrate present at the dermal-epidermal junction. Histologically, lichenoid drug eruptions are nearly identical to lichen planus, with the exception of eosinophilic infiltration and prominent parakeratosis [11].

Hence, it is possible to differentiate the two by histological comparison, although clinical correlation can be very useful [12]. This can also help in the management of the condition to prevent further breakouts, for example, by discontinuing an offending medication. For our patient, although the histological evaluation was not completed for the oral lesions, the lack of any gold-containing medications meant that it was likely an OLP, not OLL [13]. Even so, skin patch testing has not been shown to correlate with pathology diagnoses of oral lichenoid lesions. This means that a pathology evaluation is still needed to establish a complete diagnosis.

This patient’s case represents the importance of keeping local inflammatory reactions and disruptions in mind when evaluating lichen planus or other conditions brought about by immune dysfunction. While it remains to be seen whether the removal of the metal crowns will have any appreciable effect on our patient’s oral lichen planus disease course, it may be a good place to start, especially for future trials pertaining to this topic.

## Conclusions

Lichen planus can present in a multitude of locations ranging from epidermal to mucosal surfaces. Our patient with a history of atopic disease developed oral lesions with characteristic Wickham's striae. Due to her allergy panel showing allergy to gold, the testing of her dental implants revealed that they were composed of a composite alloy containing gold. Her oral lichen planus eruption was therefore likely a result of long-term mucosal irritation from the gold dental implants. This has previously been described as a potential effect of gold salt-containing medications and represents a rare presentation of how immune dysfunction can manifest unexpectedly. Patients with oral lichenoid lesions should therefore be evaluated for aggravating factors including those of dental origin.
